# An Optimized Synthetic-Bioinformatic Natural Product Antibiotic Sterilizes Multidrug-Resistant *Acinetobacter baumannii*-Infected Wounds

**DOI:** 10.1128/mSphere.00528-17

**Published:** 2018-01-24

**Authors:** Xavier Vila-Farres, John Chu, Melinda A. Ternei, Christophe Lemetre, Steven Park, David S. Perlin, Sean F. Brady

**Affiliations:** a Laboratory of Genetically Encoded Small Molecules, The Rockefeller University, New York, New York, USA; b Public Health Research Institute, New Jersey Medical School, Rutgers University, Newark, New Jersey, USA; Antimicrobial Development Specialists, LLC

**Keywords:** *Acinetobacter baumannii*, antibiotics, syn-BNP, wound infections

## Abstract

The antibiotic paenimucillin A was originally identified using a culture-independent synthetic-bioinformatic natural product (syn-BNP) discovery approach. Here we report on a bioinformatics-guided survey of paenimucillin A analogs that led to the discovery of paenimucillin C. Paenimucillin C inhibits the growth of multidrug-resistant (MDR) *Acinetobacter baumannii* clinical isolates, as well as other Gram-negative bacterial pathogens. In a rat cutaneous wound model, it completely sterilized MDR *A. baumannii* wound infections with no sign of rebound. Mechanistic studies point to a membrane-associated mode of action that results in leakage of intracellular contents.

**IMPORTANCE** Natural product-inspired antibiotics have saved millions of lives and played a critical role in modern medicine. However, the emergence of drug-resistant pathogens is outpacing the rate at which new clinically useful antibiotics are being discovered. The lack of a means to combat infections caused by multidrug-resistant (MDR) *Acinetobacter baumannii* is of particular concern. The sharp increase in cases of MDR *A. baumannii* infections in recent years prompted the CDC (https://www.cdc.gov/drugresistance/biggest_threats.html) and WHO (http://www.who.int/medicines/publications/global-priority-list-antibiotic-resistant-bacteria/en/) to list this pathogen as a “serious threat” and “critical pathogen,” respectively. Here we report a new antibiotic, paenimucillin C, active against Gram-negative bacterial pathogens, including many clinical isolates of MDR *A. baumannii* strains. Mechanistic studies point to membrane disruption leading to leakage of intracellular contents as its antibacterial mode of action. Paenimucillin C sterilizes MDR *A. baumannii* infections in a rat cutaneous wound model with no sign of rebound infection, providing a potential new therapeutic regimen.

## Abstract (precis)

Natural product-inspired antibiotics have saved millions of lives and played a critical role in modern medicine. However, the emergence of drug-resistant pathogens is outpacing the rate at which new clinically useful antibiotics are being discovered. The lack of a means to combat infections caused by multidrug-resistant (MDR) *Acinetobacter baumannii* is of particular concern. The sharp increase in cases of MDR *A. baumannii* infections in recent years prompted the CDC (https://www.cdc.gov/drugresistance/biggest_threats.html) and WHO (http://www.who.int/medicines/publications/global-priority-list-antibiotic-resistant-bacteria/en/) to list this pathogen as a “serious threat” and “critical pathogen,” respectively. Here we report a new antibiotic, paenimucillin C, active against Gram-negative bacterial pathogens, including many clinical isolates of MDR *A. baumannii* strains. Mechanistic studies point to membrane disruption leading to leakage of intracellular contents as its antibacterial mode of action. Paenimucillin C sterilizes MDR *A. baumannii* infections in a rat cutaneous wound model with no sign of rebound infection, providing a potential new therapeutic regimen.

## INTRODUCTION

Despite the widespread availability of antibiotics, infectious diseases remain the second leading cause of death worldwide and the third leading cause of death in the United States (1). Drug-resistant infections kill more than 65,000 people annually in the United States alone. The epidemiology of antibiotic resistance suggests that antibiotic resistance in the clinical setting will continue to grow as a problem for the foreseeable future. Nowhere is the need for novel antibiotics more pressing than in the treatment of Gram-negative bacterial infections. A number of recent reports have found that the absence of antibiotics capable of controlling multidrug-resistant (MDR) infections caused by the opportunistic Gram-negative pathogen *Acinetobacter baumannii* is of particular concern (2, 3). For example, the sharp increase in cases of hospital- and community-acquired MDR *A. baumannii* wound infections prompted the Centers for Disease Control and Prevention (CDC) and the World Health Organization (WHO) to call for the development of new antibiotics against MDR *A. baumannii*, listing it as a “serious threat” and “critical pathogen,” respectively (4, 5).

The majority of antimicrobials currently in clinical use are either natural products (NPs) or their synthetic derivatives (6). Unfortunately, traditional NP discovery methods provide access to only a small fraction of the bacterial biosynthetic diversity in nature as they depend on examination of metabolites produced by bacterial cultures (7, 8). New means of identifying NPs or NP-like antibiotics capable of combating antibiotic-resistant pathogens are needed to circumvent this bottleneck.

**FIG 1:**
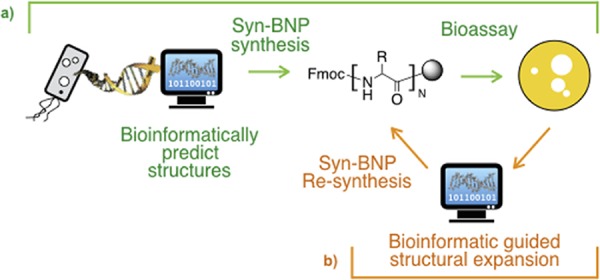
Overview of the syn-BNP approach. (a) syn-BNPs are synthesized based on a bioinformatic analysis of biosynthetic gene clusters found in sequenced bacterial genomes and assayed for bioactivities. (b) As any single bioinformatic prediction may not perfectly represent the natural product encoded by a gene cluster, additional bioinformatic analyses are used to expand the structural diversity to explore around each primary bioactive hit.

One alternative method for generating NP-like small molecules relies on the synthesis of small molecule structures bioinformatically predicted from sequenced bacterial biosynthetic gene clusters, thereby bypassing the prerequisites of culture and gene cluster expression (9, 10). The resulting synthetic-bioinformatic natural products (syn-BNPs) are not always expected to be exact mimics of NPs, but instead they represent structurally similar bioactive analogues of NPs that can serve as starting points for the development of more potent compounds (Fig. 1).

## RESULTS

**FIG 2:**
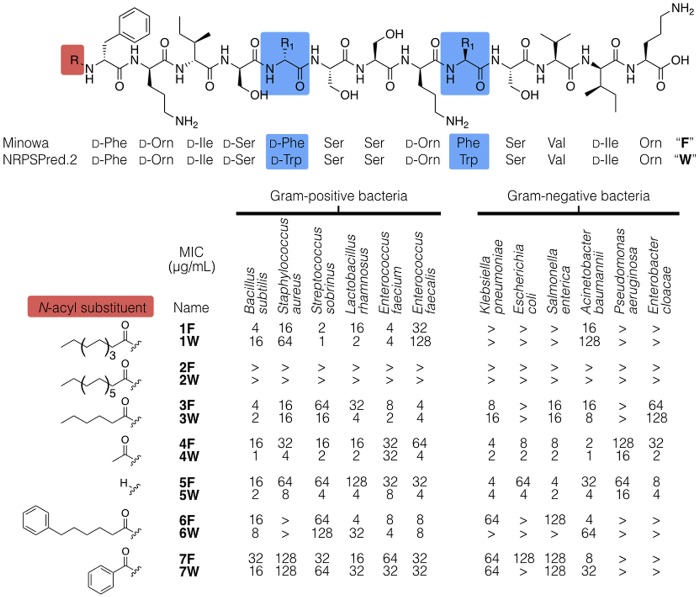
The original syn-BNPs (paenimucillin A [1F] and B [1W]) were synthesized according to Minowa and NRPSPredictor2, two algorithms commonly used for prediction of natural product structures from NRPS biosynthetic gene clusters. In this study, we systematically explored the influence of the N-terminal substituent because it is known to influence the antibacterial activity of peptides and the bioinformatic algorithms we used were not designed to provide detailed predictions of this substituent. “>” denotes a MIC of >128 μg/ml (the highest concentration tested).

We identified a series of antibiotics inspired by bioinformatic analysis of nonribosomal peptide synthetase (NRPS) gene clusters. A pair of these initial antibacterial syn-BNPs, paenimucillin A and B (1F and 1W [Fig. 2]), are closely related *N*-acylated 13-mer linear peptides containing seven d-amino acids and three nonproteinogenic residues (10). Both syn-BNPs were synthesized based on bioinformatic predictions arising from an NRPS gene cluster found in the genome of *Paenibacillus mucilaginosus* K02 (11, 12). Paenimucillin A showed broad Gram-positive antibacterial activity. Interestingly, it also showed mild antibacterial activity against the Gram-negative pathogen *A. baumannii*. The closest structure to be identified using fermentation-based NP discovery methods is the Gram-negative-active antibiotic tridecaptin. Tridecaptin and paenimucillin A share 5 of their 13 residues and an acyl substituent at their N termini (13, 14).

Although paenimucillin A (1F) only showed limited Gram-negative activity, the pressing need for antibiotics active against *A. baumannii* prompted us to explore this syn-BNP structure in more detail. Here we report the identification of paenimucillin C (4F), a potent Gram-negative active antibiotic that was inspired by a bioinformatics-guided structural expansion of the original syn-BNP, 1F. In a rat cutaneous wound model, 4F sterilized MDR *A. baumannii* infections after twice-daily treatments and the rats showed no rebound infection after 5 days.

Two commonly used bioinformatic algorithms for prediction of peptide sequence from an NRPS gene cluster (Minowa and NRPSPredictor2) (15, 16) yielded peptides that only differed by Phe-for-Trp exchanges at both positions 5 and 9. Although the bioinformatics prediction of the peptide sequence appeared robust, the identity of the N-terminal acyl substituent on an NRP is difficult to predict with certainty by bioinformatics. The N-terminal substituent on an antimicrobial peptide can profoundly influence the way it interacts with the cell envelope and hence its potency (17, 18). We therefore focused our efforts to improve the activity of 1F through the synthesis of both the Phe (F) and Trp (W) peptide series containing diverse *N*-acyl substituents. The resulting peptides span a range of hydrophilicities, with some *N*-acylated using a long-chain lipid moiety and others containing free N termini (Fig. 2).

Each new peptide was assayed for antibacterial activity against a panel of Gram-negative and Gram-positive bacteria (Table 1). The original syn-BNP, 1F, which contains a decanoyl (C_10_) *N*-substituent, is active against a number of Gram-positive bacteria we tested, including common pathogens in the *Streptococcus* genus (Fig. 2). Among the ESKAPE pathogens (*Enterococcus faecium*, *Staphylococcus aureus*, *Klebsiella pneumoniae*, *A. baumannii*, *Pseudomonas aeruginosa*, and *Enterobacter* species), it was most effective against *E. faecium* (MIC, 4 μg/ml) and showed limited activity against *A. baumannii* (MIC, 16 μg/ml). Extension of the N-terminal fatty acyl chain by four carbons (2F/W) abrogated all antibacterial activity. Similarly, replacement of the original decanoyl substituent with an aromatic moiety (6F/W or 7F/W) reduced the antibacterial potency considerably. In contrast, compounds containing shorter *N*-acyl substituents (3F/W or 4F/W) or no *N*-acyl substituent (5F/W) showed increased potency and a broader spectrum of activity.

**TABLE 1:** List of microbial strains and cell lines used in this study

Species by category	Strain	Resistance^a^
Spectrum of activity screens		
Gram positive		
* Bacillus subtilis*	168 1A1 (BGSC)	
* Enterococcus faecium*	Com15	
* Enterococcus faecalis*	Brady lab strain	V
* Staphylococcus aureus*	USA300	M
* Streptococcus sobrinus*	W1703 (BEI)	
* Lactobacillus rhamnosus*	LMS2-1 (BEI)	
Gram negative		
* Acinetobacter baumanni*	ATCC 17978	
* Enterobacter cloacae*	ATCC 14037	
* Escherichia coli*	DH5α	
* Klebsiella pneumoniae*	ATCC 10031	
* Pseudomonas aeruginosa*	PAO1	
* Salmonella enterica*	Subsp. enterica	

Cytotoxicity assessment		
* Candida albicans*	Brady lab strain	
* Homo sapiens*	HT-29 (BEI)	

Clinical isolates used in this study^b^		
* Acinetobacter baumannii*	1788	C, E, T
	1790	C, E, T
	1791	C, E, T
	1795	C, E, T
	1797	C, E, T
	S3	C, E
	S5	C, E
	AB5075	T

^a^ Resistance abbreviations: C, cephalexin; E, ertapenem; M, methicillin; T, tetracycline; V, vancomycin.

^b^ The clinical isolates used in this study are described in more detail in references 19, 20, and 22. Strains 1788 to 1797 and S3 and S5 are independent drug-resistant isolates: strains 1788 to 1797 are from the Hospital for Special Surgery (New York, NY), and strains S3 and S5 are from the clinical laboratory of Ohio State University Hospital (Columbus, OH).

The short-chain *N*-acylated structures were of particular interest to us as they gained potent activity against *A. baumannii*. MDR *A. baumannii* is a rapidly emerging concern in hospitals because of its common appearance in skin wounds and burn patients (2, 3). Both 4F and 4W are effective against all of the drug-resistant *A. baumannii* clinical isolates we tested (Table 2). Among these isolates, the S3 and S5 strains are resistant to ceftazidime and the strains 1788, 1790, 1791, 1795, and 1797 (here termed strains “1788 to 1797”) are additionally resistant to tetracycline (19, 20). These short-chain *N*-acylated analogs were not only active against *A. baumannii*, they also showed no appreciable toxicity toward the opportunistic fungal pathogen *Candida albicans* (Table 2) and no detectable hemolysis at as much as 500× their MIC. In an initial cytotoxicity assessment against a human colon cancer cell line (HT-29), 4F and 4W inhibited cell growth at 128 and 64 μg/ml after incubation at 37°C for 20 h, respectively, potentially providing a broad therapeutic window (Table 2).

**TABLE 2:** Assessment of 4F and 4W against drug-resistant clinical isolates of *A. baumannii* and its toxicity

Antibiotic	MIC (μg/ml) for:								
	*A. baumannii* clinical isolates^a^							Toxicity assessment^b^	
	1788	1790	1791	1795	1797	S3	S5	*C. albicans*	HT-29
4F	4	4	2	4	4	4	4	128	128
4W	2	0.5	0.5	2	2	4	4	128	64

^a^ All assays were performed in duplicate.

^b^ *C. albicans* and a human colon cancer cell line (HT-29) were used as surrogates to assess cytotoxicity.

**FIG 3:**
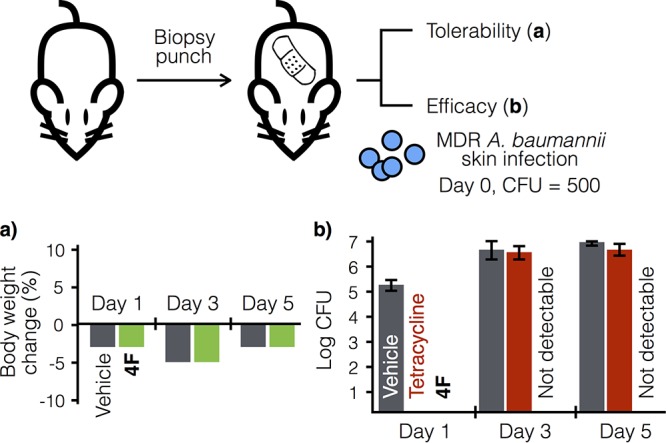
Rat cutaneous wound model study. In the tolerability assessment (a), vehicle and 4F both showed weight loss within the expected range typical of administration of analgesia (buprenorphine). The graph shows the average data tested on two independent wounds. In the efficacy assessment (b), the rats were infected on day 0 with multidrug-resistant *A. baumannii* AB5075 (500 CFU). The infected wounds were then treated twice daily, starting at 0.5 h postinoculation, with vehicle, tetracycline (10 mg/ml), or 4F (10 mg/ml). 4F completely sterilized the wound, with no rebound throughout the study. The graph shows the average data from four independent wounds per treatment per time point; error bars represent ±1 standard deviation.

The potent activity against MDR *A. baumannii* coupled with its limited toxicity led us to explore the activity of the paenimucillins in a rat cutaneous wound model. We chose to look at the activity of 4F because of its specificity against *A. baumannii* and low eukaryotic toxicity. In this study, two symmetrical 1.0-cm puncture wounds were introduced to the dorsal side muscle layer of outbred Sprague-Dawley rats (21). Vehicle control and 4F were administered topically to assess initially tolerance and potential adverse effects. Neither acute toxicity nor any change in behavior was seen, and the weight loss of 5% or less observed in this study was within the normal range due to the administration of analgesia for the wounds (Fig. 3a).

To assess efficacy, the same screening format was used, except the wounds were inoculated with MDR *A. baumannii* strain AB5075 (500 CFU) on day 0 (Fig. 3b) (22). In this study, tetracycline treatment was used as a negative control, wherein tetracycline and 4F showed *in vitro* MICs of 0.25 and 1 μg/ml, respectively. As expected with an infection by an antibiotic-resistant pathogen, after an initial reduction, the bacterial burden of the tetracycline-treated wound infection on day 3 rebounded to 6.4 ± 0.31 log CFU and showed no significant difference compared to the vehicle control (6.5 ± 0.41 log CFU). In contrast, 4F completely sterilized the wounds after 1 day, and bacterial burden remained undetectable throughout the 5-day study (0 CFU; *P* < 0.00001). Paenimucillin 4F is therefore effective at killing *A. baumannii* not only *in vitro* but also in a clinically relevant animal wound model.

**FIG 4:**
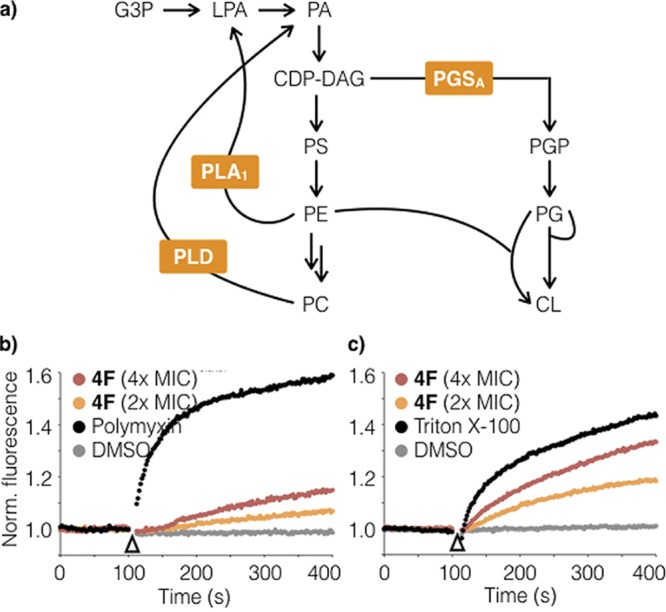
Mechanism of action studies using paenimucillin C (4F). (a) Among strains resistant to 4F, genome sequencing identified common mutations in three genes (*pla1*, *pld*, and *pgsA*) within the cell membrane phospholipid metabolism network. Abbreviations: CDP-DAG, CDP diacylglycerol, CL, cardiolipin; G3P, glycerol-3-phosphate; LPA, lysophosphatidic acid; PA, phosphatidic acid; PS, phosphatidylserine. Panels b and c show results from permeability assays using DIBAC_4_ (b) and SYTOX (c), fluorescent dyes for probing membrane depolarization and pore formation, respectively. Polymyxin and Triton X-100 serve as positive controls for membrane depolarization and pore formation, respectively.

To explore the mode of action of the paenimucillins, we attempted to raise 4F-resistant *A. baumannii* mutants. By directly plating on 4F at 2-fold its MIC, we were unable to identify any resistant mutants. Alternatively, a single *A. baumannii* ATCC 17978 colony (designated the mother strain) was grown overnight and split into five separate lineages. By daily serial passage into successively higher concentrations of 4F, we were able to identify mutants that grew in as high as 40 μg/ml of the antibiotic. All resistant strains survived in the presence of ≥20 μg/ml 4F, but grew more slowly, to a lower cell density, and had a “slimier” morphology than the parent strain, suggesting a significant fitness cost is associated with the mutations that conferred resistance. We sequenced the genomes of three independent resistant mutants from each lineage and compared the nucleotide sequences to that of the mother strain. All of these strains had acquired mutations disrupting one or more enzymes that are part of the cell membrane phospholipid metabolism network (Table 3; Fig. 4a), including phospholipase A (*pla1*), phospholipase D (*pld*), and a phosphatidyltransferase (*pgsA*). The only other mutation that was found in multiple sequenced genomes was in a sensor histidine kinase gene (*adeS*).

**TABLE 3:** Common mutations acquired by *A. baumannii* strains resistant to 4F

Mutation in lineage^a^:					Gene	Product
1	2	3	4	5		
Χ	Χ	Χ	Χ	Χ	*pla1*	Phospholipase A1
	Χ	Χ			*pld*	Phospholipase D
		Χ^b^	Χ	Χ	*adeS*	Sensor histidine kinase
Χ	Χ	Χ			*pgsA*	CDP-diacylglycerol-glycerol-3-phosphate 3-phosphatidyltransferase

^a^ This table lists only those mutations that were acquired by two or more lineages. Three strains were sequenced per lineage. The symbol “Χ” denotes genes that are disrupted in all three sequenced strains within a lineage.

^b^ Two out of three sequenced strains in lineage 3 show mutations in the *adeS* gene.

Phospholipase A1 is an esterase that catalyzes the hydrolysis of the fatty acyl chain on the sn-1 position of phosphatidylethanolamine (PE), and phospholipase D hydrolyzes phosphatidylcholine (PC). The *pgsA* gene encodes an enzyme responsible for the synthesis of phosphatidylglycerol phosphate (PGP), which is the precursor of phosphatidylglycerol (PG) synthesis. These mutations, which are all associated with phospholipid metabolism, likely each result in the reduction of PG and an increase of PE and PC (Fig. 4a) (23, 24). Alterations in the ratio of phospholipid components in the cell membrane or the enzymes that influence phospholipid component ratios—in particular, phospholipases A1 and D in Gram-negative bacteria—have been linked to changes in antibiotic resistance and pathogenicity (25–27).

Three out of the five resistant mutant lineages also acquired a mutation in the *adeS* gene. AdeS is the histidine kinase of a two-component regulatory system, which modulates the activity of AdeR in response to environmental cues. AdeR controls the expression of the AdeABC drug efflux pump (28, 29). Overexpression of *adeABC* is known to change the composition of membrane proteins in *A. baumannii* and leads to impairment in membrane-associated functions—e.g., cell adhesion and biofilm formation (30).

In addition to the mutations mentioned above, lineages 1 and 5 each acquired a mutation in *lpxD* and *lpxA*, respectively, both of which code for enzymes that are part of the lipopolysaccharide biosynthesis pathway. These resistance mutations we detected either directly disrupt enzymes that are part of the cell membrane phospholipid metabolism network or indirectly lead to altered membrane phospholipid composition, which suggested the disruption of membrane integrity as a potential mechanism of action.

The effect of 4F on membrane integrity was assessed using DIBAC_4_ and SYTOX, fluorescent dyes that report on membrane depolarization and pore formation, respectively (31, 32). In these studies, 4F was added at 2× and 4× its MIC (4 or 8 μg/ml) and its activity was compared to that of the positive controls polymyxin (4× MIC) and Triton X-100 (1% [wt/vol]), respectively. In the membrane depolarization assay, exposure to 4F at 4× its MIC caused moderate depolarization, albeit to a much lesser extent relative to polymyxin (~25% at the endpoint of the assay). In contrast, at the same concentration, 4F resulted in pore formation comparable to (~75%) that of Triton X-100. It appears that 4F likely exerts its antibacterial effect by causing cytoplasmic content leakage. Interestingly, tridecaptin, the closest natural product structure to the paenimucillins, was recently shown to target lipid II of Gram-negative bacteria without causing membrane leakage or depolarization (33). It is noteworthy that the potency of 4F varies considerably across bacterial species (Fig. 2), suggesting that it targets the membrane of *A. baumannii* in a distinct fashion. Additional experiments will be required to elucidate the mechanistic details of its mode of action. The membrane-targeting mechanism of action of 4F and its net positive charge (3+) at neutral pH are typical features seen in cationic peptide antibiotics. Its low cytotoxicity and low resistance development are reminiscent of a growing list of cationic peptides that are in various stages of clinical trials (34). These data suggest that 4F represents a new lead structure that warrants additional exploration toward the development of new treatments for MDR *A. baumannii* wound infections.

## DISCUSSION

Our bioinformatics-guided structural expansion study of 1F focused on the N-terminal substituent, as it is difficult to predict bioinformatically. The resulting new antibiotic, 4F, is more potent, shows a broader spectrum of activity, and is active in sterilizing cutaneous wound infections of MDR *A. baumannii* in a rat model. From its inception, synthetic chemistry has drawn inspiration from natural products for the synthesis of bioactive molecules. We see the syn-BNP approach as providing an alternative means for deriving synthetic inspiration from nature. In the case of syn-BNPs, the inspiration is derived from bioinformatic analyses of primary sequences from microbial genomes instead of chemical analyses of microbial metabolites from fermentation cultures. With the ever-growing number of sequenced bacterial genomes and the continuous improvement of bioinformatics algorithms for prediction of chemical structures from biosynthetic gene clusters, we believe the syn-BNP approach will prove increasingly useful as a tool for identification of bioactive small molecules from nature.

## MATERIALS AND METHODS

### Instruments, materials, media, and consumables.

Wang resins were purchased from Matrix Innovation. Reagents for peptide synthesis were purchased from P3 BioSystems, Chempep, and Chem-Impex International. The fluorescent dyes DiBAC_4_ and SYTOX were purchased from Thermo Fisher Scientific, and media were prepared from premixed powders: brain heart infusion for *S. sobrinus* and *L. rhamnosus* (Hardy Diagnostics), yeast extract-peptone-dextrose for *C. albicans*, lysogeny broth (LB) for all other bacteria (BD Biosciences), and McCoy’s 5A medium with 10% fetal bovine serum for HT-29 cells (American Type Culture Collection). All other chemicals and consumables were purchased from Sigma-Aldrich and VWR International. Paenimucillin 4F for *in vivo* studies was purchased from Ontores Biotechnologies as a high-performance liquid chromatography (HPLC)-purified peptide and used as is. Dye assays were performed in a Gen5 microtiter plate reader (Biotek Instruments). Liquid chromatography-mass spectrometry (LC-MS) analyses were performed by using an Acquity system (Waters Corporation). The microbial strains used and cell line information are listed in Table 1.

### Peptide synthesis.

*N*α-9-Fluoroenylmethoxy carbonyl (Fmoc)-*N*δ-Boc-l-ornithine (3.2 mmol) was activated by *N*,*N*′-diisopropylcarbodiimide (DIC; 1.6 mmol) in a 2:1 mixture of dichloromethane-dimethylformamide (DCM-DMF; 25 ml) and then added to Wang resin (0.4 mmol) in the presence of *N*,*N*-dimethylaminopyridine (DMAP; 0.04 mmol). The slurry was shaken at room temperature for 2 h, and the loading yield was quantitated based on the amount of Fmoc adduct resulting from piperidine microcleavage. The peptide portion of the paenimucillins was synthesized in bulk (200 μmol) using an automated microwave peptide synthesizer (Biotage Alstra Initiator^+^). For Fmoc deprotection, the resin was treated twice at 75°C for 3 min with 20% piperidine in DMF (4.5 ml). For peptide coupling, the resin was mixed with amino acid (3 eq), PyBOP (3 eq), Cl-HOBt (9 eq), and diisopropylethylamine (DIEA; 9 eq) in DMF (4.5 ml) and heated to 75°C for 10 min. The resin was washed four times with DMF (9 ml) after each deprotection and coupling step. Resins containing crude peptides were then split into smaller aliquots (40 μmol) and coupled manually to various *N*-substituents. In manual syntheses, Fmoc groups were removed by treating the resin twice with 20% piperidine in DMF (4.5 ml) at room temperature for 10 min. During each coupling step, the *N*-substituent (3 eq) was activated by mixing with PyBOP (3 eq), Cl-HOBt (9 eq), and DIEA (9 eq) in DMF (4.5 ml). The activated *N*-substituent was then added to the resin and allowed to react for 1 h at room temperature. The coupling step was repeated to drive the reaction to completion. Upon completion of syntheses, resin-bound peptides were cleaved by a trifluoroacetic acid (TFA) cocktail supplemented with 2.5% (vol/vol) of each of water and triisopropylsilane (TIPS). The cleavage solution was then added into cold *tert*-butyl methyl ether, and the precipitate was redissolved in 50% MeCN for HPLC purification. All purified peptides were verified by high-resolution mass spectrometry. Paenimucillin C (4F) was additionally characterized by ^1^H- and ^13^C-nuclear magnetic resonance (NMR) spectroscopy.

### Hemolytic assay.

The hemolytic assay was performed on tryptic soy agar plates embedded with 5% sheep blood. A series of 25% (vol/vol) dimethyl sulfoxide (DMSO) solutions containing 8, 4, 2, and 1 mg/ml of 4F or 4W were prepared, corresponding to 4,000×, 2,000×, 1,000×, and 500× MIC. Each solution (5 μl) was spotted on premarked locations on the blood agar. After the solution had been absorbed, the plates were incubated at 30°C for 2 days. Triton X-100 (0.4% [wt/vol]) and 25% DMSO were used as the positive and negative controls, respectively. Discoloration is indicative of hemolysis.

### Susceptibility and cytotoxicity assays.

Standard susceptibility assays were performed in LB medium in 96-well microtiter plates to determine the MIC by the broth microdilution method in accordance with protocols recommended by Clinical and Laboratory Standards Institute (35). Cytotoxicity was assessed using human HT-29 cells based on a similar broth microdilution method (36). All assays were performed in duplicate. For the cytotoxicity assay, 5,000 HT-29 cells per well were seeded into a microtiter plate and cultured for 24 h at 37°C in a 5% CO_2_ atmosphere. Peptides were supplemented at concentrations ranging from 128 to 0.5 μg/ml, and the cells were incubated for another 20 h. Growth media were removed, 3-(4,5-dimethylthiazol-2-yl)-2,5-diphenyltetrazolium bromide (MTT) was added to all wells (220 μl per well at 0.5 mg/ml), the mixture was incubated for 4 h, and then the media were discarded. Cell viability was determined by visual inspection.

### Membrane integrity assays.

In a membrane depolarization assay (31), *A. baumannii* colonies were scraped off an agar plate and suspended in LB to optical density at 600 nm (OD_600_) of 0.5 to 0.6. The resulting suspension (1.8 ml) was mixed 9:1 with DiBAC_4_ (20 μg/ml), incubated at room temperature for 5 min, and monitored at 2-s intervals (excitation/emission, 492/515 nm) for 2 min. Various solutions were added (10 μl) while the fluorescence signal was continually being monitored. Paenimucillin C was tested at final concentrations of 2× and 4× its MIC, and polymyxin B and DMSO were used as positive and negative controls, respectively. The treated cultures were then monitored at 2-s intervals for another 5 min. The raw fluorescence signals normalized relative to the DMSO-treated negative control are reported in Fig. 4b. Pore formation assays (32) were performed in the same way, except that Triton X-100 was used as the positive control at a final concentration of 0.25% (wt/vol).

### Raising resistant mutants.

A single *A. baumannii* ATCC 17978 colony (designated the mother cells) from a freshly streaked plate was inoculated into LB medium and grown overnight at 37°C. To raise resistant mutants by direct plating, the overnight mother culture was diluted 200-fold, supplemented with syn-BNP (4F) at 4 μg/ml (2× MIC), and distributed into 480 individual microtiter wells (100 μl per well). No resistant colonies were observed after static incubation at 37°C for 24 h. To raise resistant mutants by serial passage, five lineages were generated by inoculating separate LB media (4 ml for each lineage) containing 2 μg/ml of 4F each with 20 μl of the overnight mother culture. The inoculated cultures were grown shaken (200 rpm) at 37°C for 24 h and, on the next day, used to inoculate LB media containing the next higher concentration of 4F in the following order: 2, 4, 8, 12, 16, 20, 24, 28, 32, 36, and 40 μg/ml. Glycerol stocks were kept for all generations for all lineages; the experiment was terminated after the 40-μg/ml selection (10× the MIC of the mother). The resulting resistant strains were subjected to whole-genome sequencing and cross-resistance screens.

The mother cells and three random colonies that sustained the highest concentration of 4F in each lineage were selected for genome sequencing. Genomic DNA extractions were performed based on published protocols. The genomes were then bar-coded, amplified, and sequenced using MiSeq based on instruction manuals provided by the manufacturer (Illumina). De-barcoded reads were assessed for mutations by comparing each read against the reference genome of *Acinetobacter baumannii* ATCC 17978-mff (GenBank accession no. NZ_CP012004.1). Differences between the mother cell and the reference genome were subtracted from those observed in the offspring lineages that show resistance to 4F.

### Rat cutaneous wound model.

The rat cutaneous wound model was used as described previously (21). Protocols for animal studies were approved by the Rutgers University Institutional Animal Care and Use Committee. All animals were humanely euthanized at the study endpoint by following the *AVMA Guidelines for the Euthanasia of Animals*. Immunocompetent Sprague-Dawley female rats (~200 g) were anesthetized by intraperitoneal injection of 100 mg/kg ketamine plus 10 mg/kg xylazine. Using a 1.0-cm-diameter disposable biopsy punch, two symmetrical wounds were created on the dorsum of each rat. A circular gauze dressing 8 mm in diameter was saturated overnight with either vehicle or 4F at 10 mg/ml in sterile centrifuge tubes. To assess tolerability, the rats were weighed daily and observed for wound healing, behavior changes, and visible adverse drug effects for 5 days. To assess efficacy, *A. baumannii* strain AB5075 was grown overnight and the culture was diluted to provide the challenge inoculum (500 CFU) as 50 μl of bacterial suspension in 0.9% (wt/vol) NaCl. A circular gauze dressing 8 mm in diameter was saturated overnight with vehicle, tetracycline, or 4F at 10 mg/ml in sterile centrifuge tubes. These saturated dressings were topically applied to the wound site with sterile forceps twice daily, starting at 30 min postinoculation, for 6 days. Rats were observed twice daily for morbidity and possible signs of acute toxicity. Two and four independent cutaneous wounds were tested in tolerability and efficacy assessments, respectively.
